# Changes in the Age-Adjusted Rate of Older Adults Dying From a Fall and Reporting a Fall and Fall Injury, 2012–2018

**DOI:** 10.1093/geroni/igaa057.2798

**Published:** 2020-12-16

**Authors:** Elizabeth Burns, Ramakrishna Kakara, Briana Moreland, Ankita Henry

**Affiliations:** 1 Centers for Disease Control and Prevention, Atlanta, Georgia, United States; 2 CDC, Atlanta, Georgia, United States; 3 Synergy America Inc., Atlanta, Georgia, United States

## Abstract

Falls are a leading cause of injury among older men and women (≥65 years) in the United States. Vital Statistics and Behavioral Risk Factor Surveillance System data were analyzed to determine the age-adjusted fall death rate, the rates of older adults reporting a fall and fall injury, and associated trends. The fall death rate increased 16% from 55.3/100,000 in 2012 to 64.4/100,000 in 2018 (p≤0.05). Like the rates in 2012, the rate of falls reported in 2018 was 713/1000 older adults and the rate of fall injuries reported was 171/1000 older adults. When assessing the rates of older adults reporting a fall or fall injury by sex, the rates among men increased from 2012 to 2016 from 637/1000 to 773/1000 (21% increase, p≤0.05) for falls and from 120/1000 to 153/1000 (28% increase, p≤0.05) for fall injuries. Understanding how these data change over time can inform targeted interventions to reduce falls.

